# Co-design of experimental nature-based solutions for decentralized dry-weather runoff treatment retrofitted in a densely urbanized area in Central America

**DOI:** 10.1007/s13280-020-01457-y

**Published:** 2021-02-07

**Authors:** Maria Pérez Rubi, Jochen Hack

**Affiliations:** Technische Universität Darmstadt, Institute of Applied Geosciences, Section of Ecological Engineering, Research Group SEE-URBAN-WATER, Schnittspahnstr. 9, 64287 Darmstadt, Germany

**Keywords:** Co-design, Dry-weather runoff, Green infrastructure, Nature-based Solutions, Retrofit

## Abstract

The quality of water in many urban rivers in Latin America is increasingly degrading due to wastewater and runoff discharges from urban sprawl. Due to deficits in sanitary drainage systems, greywater is discharged to the stormwater drainage network generating a continuous dry-weather runoff that reaches rivers without treatment. One of the main challenges in the region is to achieve sustainable management of urban runoff for the recovery of rivers ecosystem integrity. However, retrofitting conventional centralized wastewater drainage networks into the existing urban grid represents important social, economic and technical challenges. This paper presents an alternative adaptive methodology for the design of Nature-based Solutions for decentralized urban runoff treatment. Through this study, technical solutions commonly used for stormwater management were adapted for dry-weather runoff treatment and co-designed for the particular conditions of a representative study area, considering space availability as the main constraining factor for retrofitting in urban areas. The application of a co-design process in a dense neighbourhood of the Great Metropolitan area of Costa Rica brought to light valuable insights about conditions that could be hindering the implementation of NBS infrastructures in Latin America.

## Introduction

A prevailing problem in developing countries of Latin America is water quality degradation of water bodies (IANAS 2019). Rivers are recipients of wastewater discharge with none or insufficient treatment (OMS/UNICEF 2016). Water quality degradation prevails both in rural and urban areas, nevertheless, pollution levels become critical in urban areas where population density is higher and larger volume of wastewater and stormwater runoff is discharged in shorter sections of the rivers (Michaud et al. 2012; Mondragón-Monroy and Honey-Rosés 2016). Despite water quality degradation and hydraulic stress, urban rivers in Latin America mostly exhibit low hydromorphological changes and well preserved riparian areas (Bergoeing 2013). These facts point out that effort should be addressed toward sustainable management of wastewater and urban runoff to recover river ecosystems.

Centralized wastewater treatment technologies in Latin America are typically implemented under top-down approaches based on standardized guidelines with merely technical data from developed countries; representing technical and economic challenges for their application. E.g., Investments of millions of US dollars have been budgeted by the national water utility in Costa Rica to build a treatment plant and retrofit a sanitary drainage system into the existing dense urban grid of San Jose (Instituto Costarricense de Acueductos y Alcantarillado 2017a, b). In Costa Rica, water supply networks cover more than 94% of the country; however, only 10% of wastewater is treated before discharged to rivers (Instituto Costarricense de Acueductos y Alcantarillados 2018). Areas lacking sanitary drainage system implement domestic septic tanks for black water disposal (i.e. toilet flushing) and discharge greywater (i.e. wastewater from kitchens, washing machines, showers, sinks) into the stormwater drainage system, producing a continuous dry-weather runoff.

Throughout this research we explore the application of an alternative process to co-design (Polk 2015; Webb et al. 2018; Wilk et al. 2020) decentralized technologies for dry-weather runoff treatment in urbanized areas. Despite the fact that dry-weather runoff treatment is not a mainstream research topic, innovative approaches for sustainable stormwater management shed a light on urban runoff treatment. These innovative approaches focus on the treatment of runoff as close to the source as possible, integrating quantity and quality control in a sustainable manner (Fletcher et al. 2015; Ahammed 2017; Raspati et al. 2017). Different terminology is being used for sustainable stormwater management research and practices around the world (Fletcher et al. 2015), such as Water Sensitive Urban Design (WSUD), Low Impact Development (LID), and Sustainable Urban Drainage Systems (SUDS). Under these concepts, infrastructural measures such as bioswales, wetlands, bioretention, and bioinfiltration systems have proven to improve water quality of stormwater runoff (Liquete et al. 2016; Irvine and Kim 2018; Purvis et al. 2018). They promote natural processes such as infiltration, evapotranspiration, conveyance, retention, and detention of runoff using the urban landscape features; thus, they can be considered Nature-based Solutions (NBS).

The aim of this research is to develop an experimental co-design process to propose solutions for dry-weather runoff treatment in dense urban areas, expecting that these solutions are better context-adapted than conventional centralized systems. A transdisciplinary approach (Lang et al. 2012; Nicolescu 2012; Polk 2015; Hoffmann et al. 2017; Femenías and Thuvander 2018) led the co-design process to understand the complexity of real-world problems in the attempt of producing holistic sustainable solutions. Throughout the co-design process, nature-based infrastructures commonly used for stormwater management were adapted for dry-weather runoff treatment, after analysing three factors of site-specific conditions in a case study area. The co-design process sought to include participatory and stakeholder based knowledge, by integrating multiple disciplines and lived-experience of local dwellers. This paper describes the application of the methodology in a neighbourhood of the Great Metropolitan Area of Costa Rica; that serve as a real-world laboratory (Evans and Karvonen 2016; Parodi et al. 2017; Wanner et al. 2018). The scope of this article is the co-design process and the implementation of experimental prototypes, to draw conclusions about real-world challenges of designing and implementing retrofitted NBS for urban runoff management in developing countries of Latin America.

## Materials and methods

Two key tenets were set to carry out this research - retrofitting and co-design. Retrofitting in this context is the process of adaptation, modification or addition of features to existing infrastructure of the urban landscape with the purpose of improving water quality of urban runoff before it is discharged to the river. The focus of this research was to design solutions for fully developed urban areas, where the installation of wastewater drainage and treatment systems was not considered during the planning phase. Therefore, retrofitting is the only option to improve the condition of the wastewater without treatment reaching natural water bodies, whilst minimizing extensive changes to the existing urban grid.

Co-design is a transdisciplinary process that involves a range of different stakeholders in the creation, redesign, or evaluation of a service or product (Polk 2015; Webb et al. 2018; Wilk et al. 2020). Experts and end-users are encouraged to participate in the process in order to combine professional expertise and lived-experience in problem solving. There are no fixed step-by-step procedures for co-design applications.

### Co-design process

In this research, the application of co-design was achieved by engaging people who were potential users and/or likely to be impacted by the outcomes. In this case, local dwellers played an essential role in the co-design since they are the final users of any proposed solution. Therefore, they were considered experts in their own experience and perception. Professional experts were also engaged during the process, these were representatives of the local government, a water supply company, central government institutions, and universities. The research was conducted within a transdisciplinary framework; the contributions of different disciplines and non-scientific knowledge from locals were systematically integrated throughout the process.

**Fig. 1:**
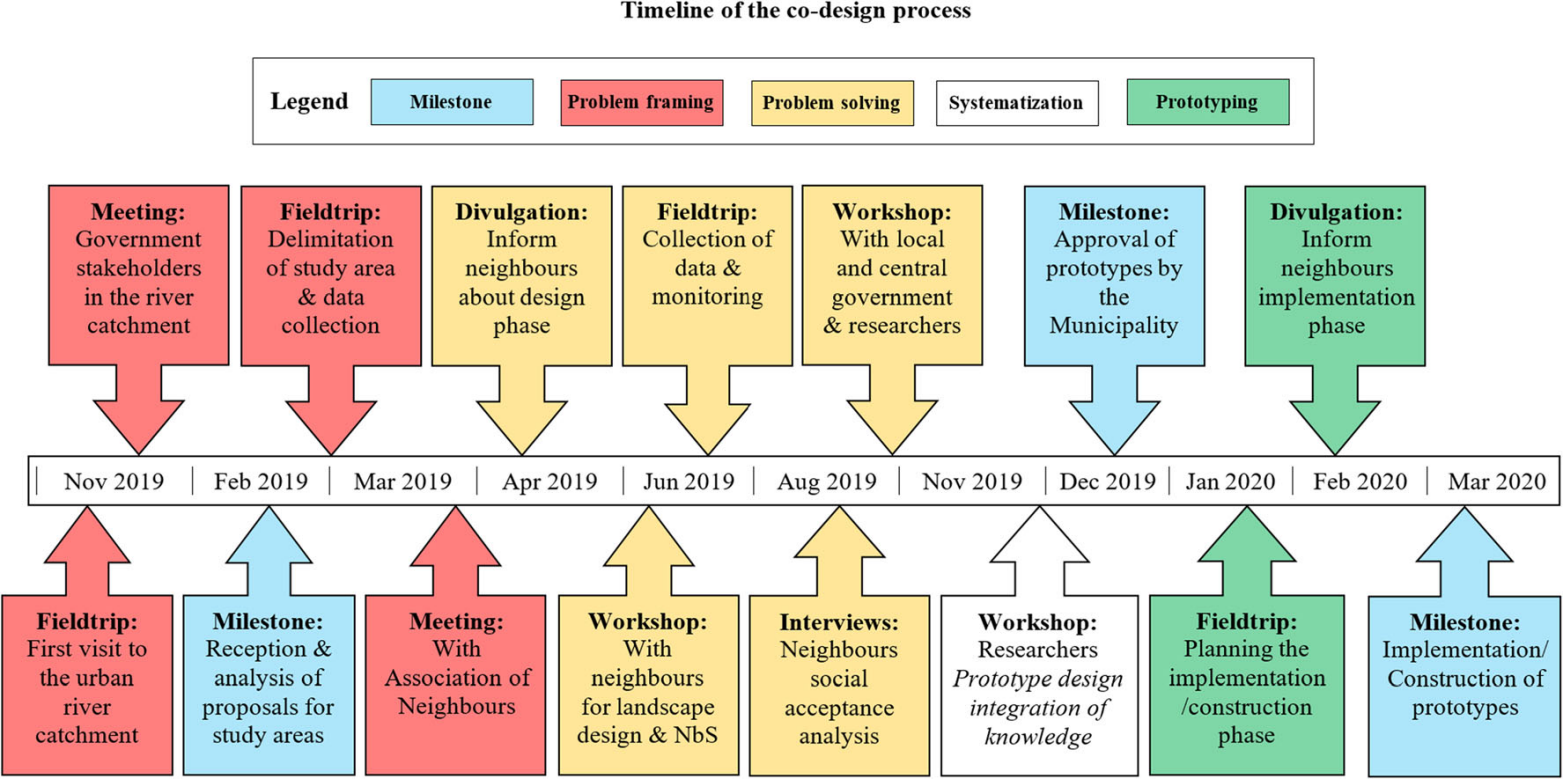
Timeline of the co-design process implemented throughout this research. The process started by the end of 2018, red boxes show activities related to problem contextualization; yellow boxes show activities for the collection of information and analysis for potential solutions; green boxes show activities for the implementation of prototypes; blue boxes show milestones along the process; the only white box shows the synthetisation of information for prototypes design and proposal

Figure 1 shows the progress, activities and participants of the co-design process. Different stakeholders were engaged with the objective of collecting, analysing, and synthetizing information about the local problem situation (activities in red boxes in Fig. 1) and insights about key aspects for potential solutions (activities in yellow boxes in Fig. 1). Field visits were the initial step for problem identification and understanding. We started the actor engagement process primarily with government stakeholders in the river catchment, aiming to understand the background and broadest contextual perspective for the problem framing; furthermore, we considered them potential owners and/or enablers of solutions. For that reason, we involved them in the selection of a study area, by asking them to propose potential sites based on their experience and knowledge.

After the case study area was selected, we started engaging local community stakeholders. As daily users of NBS to be implemented, the neighbours provide relevant insights on how they perceive their surroundings and how they use it or want to use it in the future. This information contributed to adapt the NBS design to the local context. These stakeholders brought their own perceptions and priorities for problem solving and we considered them potential collaborative partners and resource providers for the subsequent implementation phase. Simultaneously, the transdisciplinary team collected information on biophysical factors of the study area. The proposal of NBS prototypes was completed during an interdisciplinary workshop with researchers of this project to synthetize all the information gathered (white box in Fig. 1) after one year of starting the co-design process. The municipality gave approval to construct them as experimental field-scale prototypes, launching the implementation phase of the project (green boxes in Fig. 1).

### Knowledge integration

**Fig. 2:**
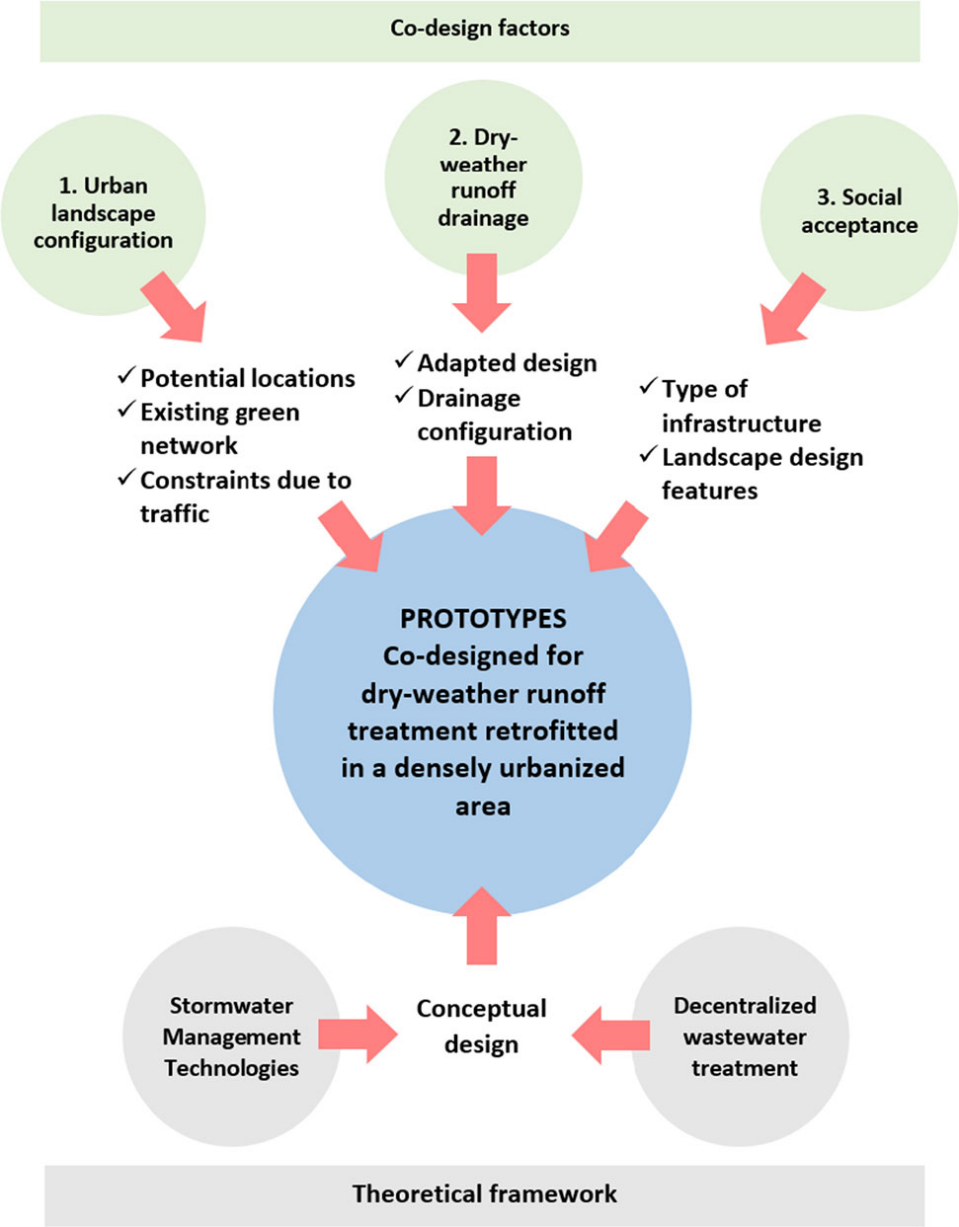
Diagram of the methodology implemented to co-design retrofit Nature-based Solutions for dry-weather runoff treatment. Three main factors were analysed (green elements in the figure); i.e. urban landscape configuration, dry-weather runoff drainage, and social acceptance. Literature references about decentralized nature-based wastewater treatment and stormwater management technologies were consulted (grey elements in the figure); served as the theoretical framework for the adaptation of infrastructure to the site-specific conditions

The method developed in this paper consists in the integration of non-scientific knowledge regarding three main factors of the area of intervention, shown as green elements in Fig. 2; with scientific knowledge of existing technologies for water quality improvement, shown as grey elements in Fig. 2. A transdisciplinary approach was implemented to retrieve knowledge from the area of intervention. Grey elements are strictly theoretical, based on international and local guidelines and regulations; this was not considered part of the transdisciplinary process. The integration consisted in the adaptation of stablished infrastructure designs to fit in the limited space available in the area of intervention whilst performing socially prioritized functions.

The three main factors of the area of intervention analysed during the co-design process to retrofit solutions (green elements in Fig. 2) were the following. (1) The configuration of the existing urban landscape. Information regarding public space (i.e. roads, sidewalks, parks) and private parcels was collected in order to identify the extent, quality and functions of existing network of green spaces or corridors. (2) The configuration of the existing drainage infrastructure that defines the occurrence and pathway of dry-weather runoff. The objective of analysing these two factors was to identify potential space for the implementation of prototypes and to integrate them into the existing green network without compromising the main function of roads or other landscape components, minimizing severe changes to the existing urban landscape and drainage configuration. The third factor considered for the co-design (3), was social perception and residents’ acceptance of innovative technology for the treatment of greywater near the source (next to their properties). The information about these site-specific factors, considered for the co-design process, was collected during field trips to the study area through interviews, meetings with authorities, workshops and field visits.

During the literature review, we retrieved information about nature-based decentralized wastewater treatment and stormwater management infrastructures (grey elements in Fig. 2). In addition, we consulted national regulations and guidelines regarding road typologies and designs, public space, and urbanization.

The implemented methodology intended to narrow the most important factors to be considered in the co-design of decentralised technologies for dry-weather runoff treatment retrofitted in densely urbanized areas and was experimentally applied in a case study area, described in the following sections.

### Study area

**Fig. 3:**
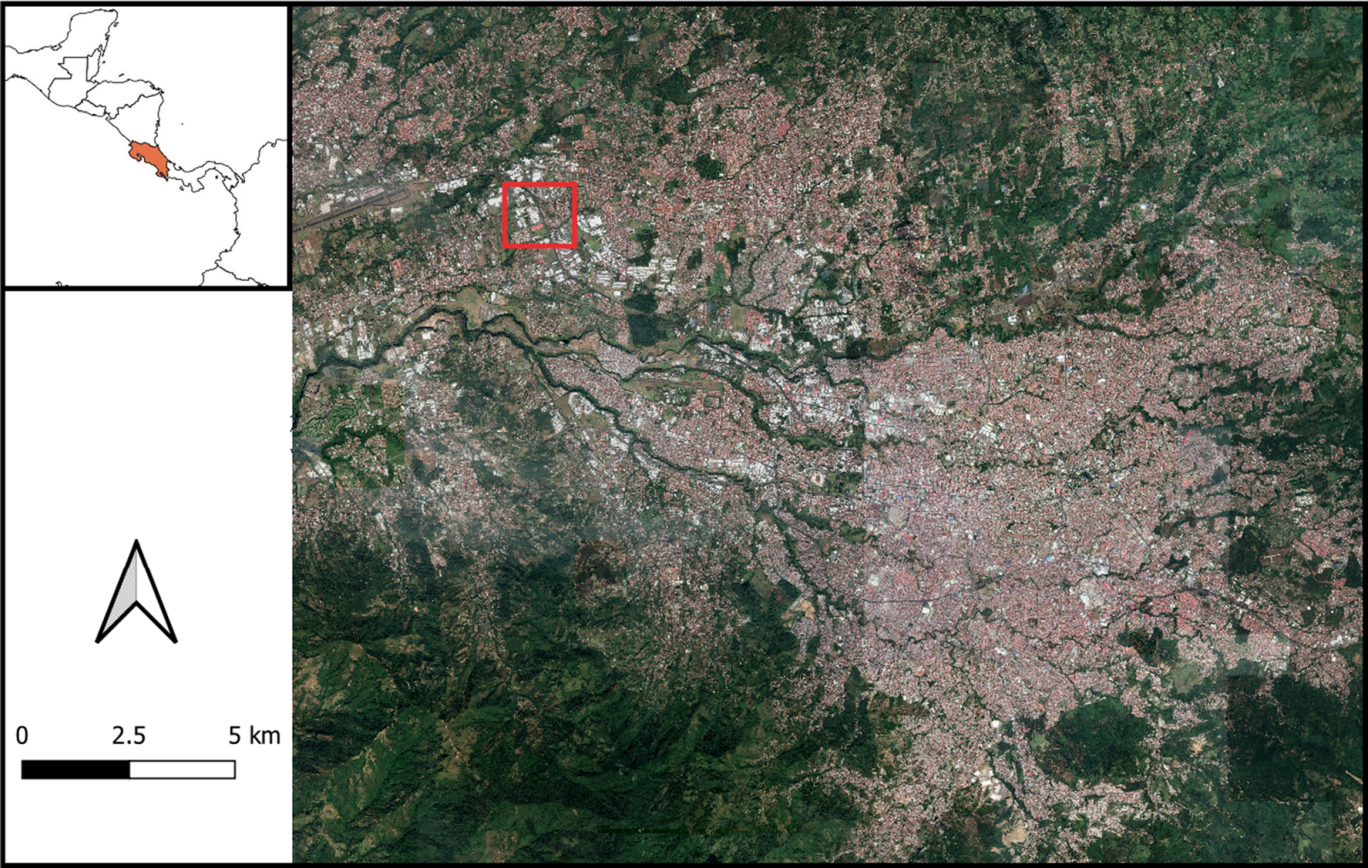
Location of the study area; neighbourhood Siglo XXI, Llorente, Flores; in the Great Metropolitan Area of Costa Rica. Source: José Fernando Chapa

To develop this research, a representative case study area in Costa Rica was selected. The prioritized features for the selection of the study area were tropical conditions, dense urbanization, occurrence of dry-weather runoff and spatial connection with river ecosystems. The Great Metropolitan Area (GAM, Spanish acronym) of San Jose is an urban sprawl in the central valley of the country facing pollution of rivers, deficient or inexistent sanitary drainage infrastructure, traffic congestion and mismanagement of solid waste and wastewater (Calvo Brenes and Mora Molina 2012; Pujol-Mesalles and Molina 2013; Mena-Rivera et al. 2017); these problems are representative of other urban developments in Central America. Within the GAM, a densely urbanized neighbourhood named “Siglo XXI” in the District of Llorente in the Canton of Flores is the selected area for the establishment of a real-world laboratory to co-design and implement experimental prototypes for dry-weather runoff treatment; its location is shown in Fig. 3.

Continuous dry-weather runoff (i.e. greywater) flowing through the stormwater drainage infrastructure is discharged without any treatment to the river known as Quebrada Seca-Burio, located at the south border of the neighbourhood (Fig. 3). Problems in this river’s catchment are hydraulic stress, riverbank instability and scouring, river water quality degradation, solid waste disposal, flood vulnerability, and loss of green areas due to urban intensification; problems that are common to most of the river catchments in the GAM (Oreamuno Vega and Herrera 2015; Mena-Rivera et al. 2017).

#### Co-design factor 1: urban landscape configuration (Fluhrer et al. 2021)

**Fig. 4:**
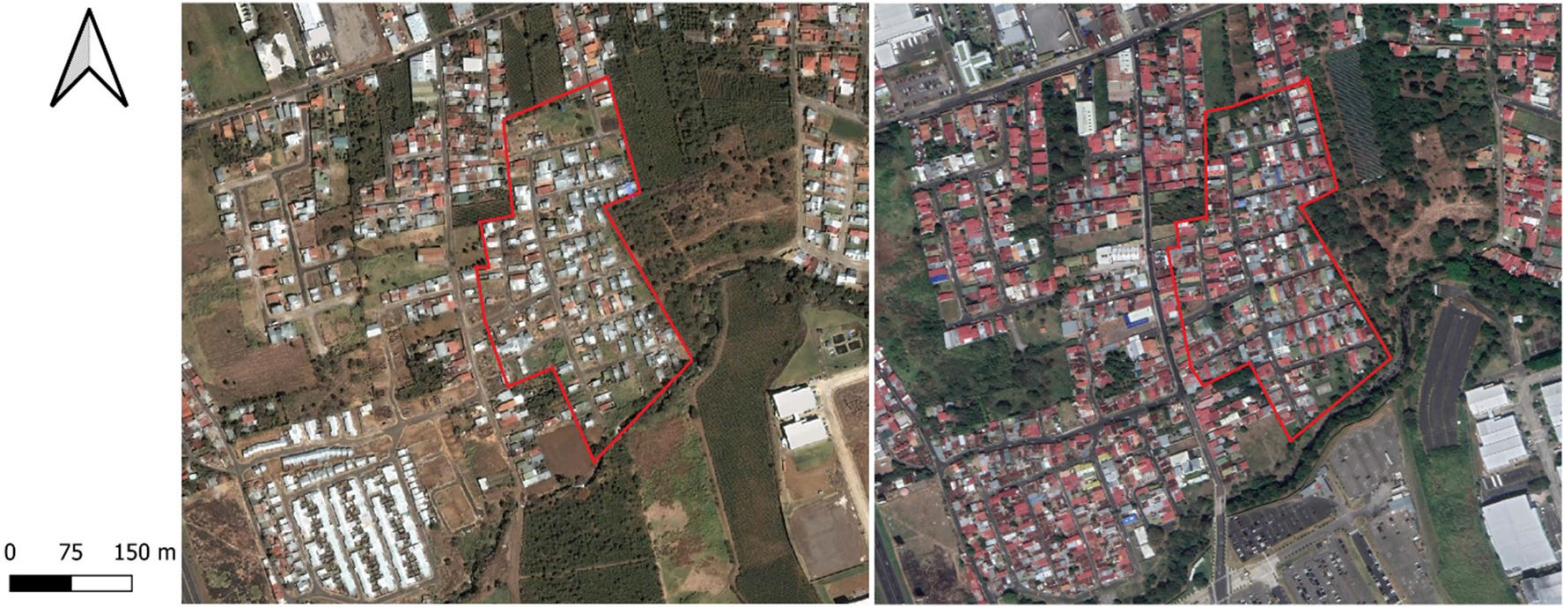
Urbanization progress in the study area, marked in red. Image on the left shows the area in 2003; the image on the right shows the dense urban grid as of January 2020. Source: José Fernando Chapa

The study area is merely residential; only two small commercial establishments are located within the boundaries of the neighbourhood, a small bakery and a convenience store. The low-income neighbourhood has a total area of 0.12 km^2^; it has been undergoing rapid densification and loss of green areas during the last two decades, as depicted in Fig. 4. Nowadays, impermeable areas account for about 57% i.e. buildings and streets; vegetated areas account for about 18% and vacant land 21%. Properties consist of single-family detached homes, arranged in narrow quadrants with an average area of 120 m^2^, in the majority of cases the property is fully built consisting of one or two-story buildings. Foreyards of properties are mostly sealed surfaces used as garage for cars.

The landscape configuration was analysed by carrying out several field visits to the study area, doing area measurements, observing the behaviour of neighbours with respect to public areas, analysing satellite images and interviewing members of the municipality, who provided the cadastral data for the neighbourhood.

#### Co-design factor 2: dry-weather runoff drainage

**Fig. 5:**
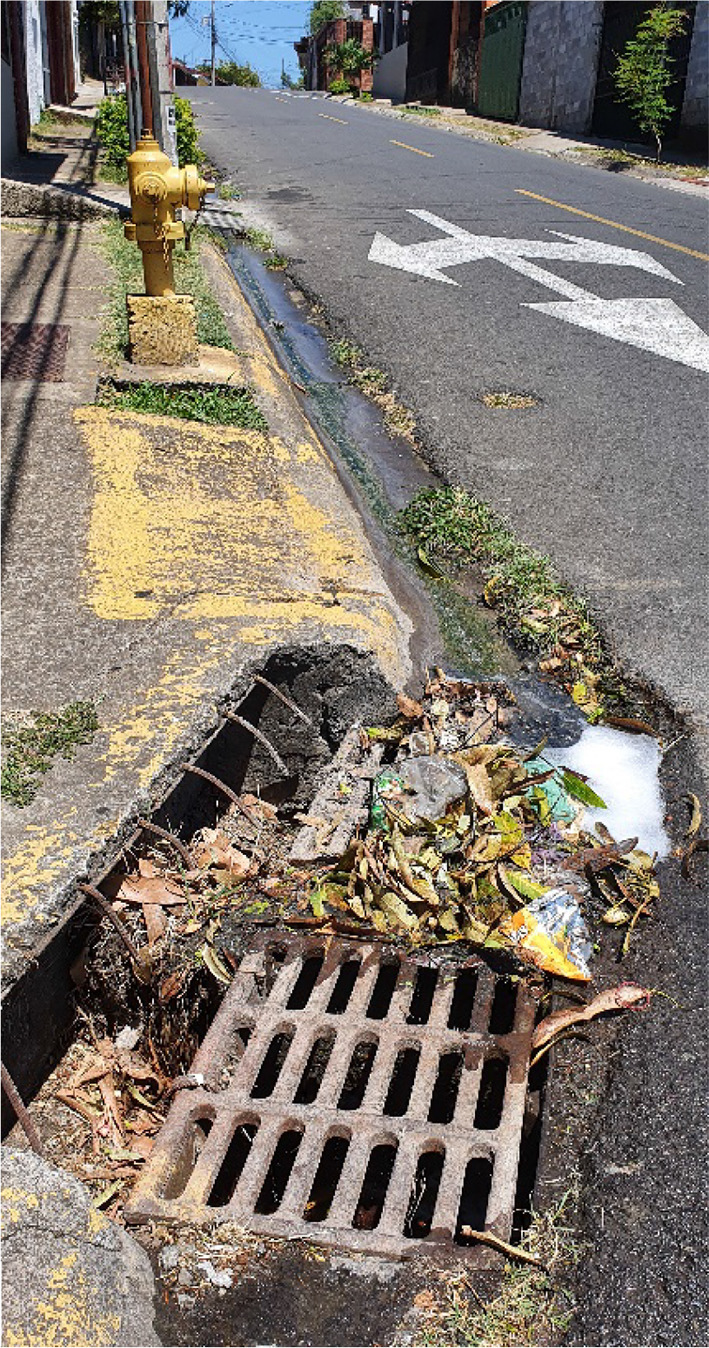
Dry-weather runoff in Siglo XXI, runoff consists on households’ greywater discharged to the gutter of streets. Source: The author, Jan. 2020

Continuous dry-weather runoff is characteristic of the study area. The water supply network covers 100% of the area, with a system of wells, storage tanks, and distribution networks owned and operated by the Municipality of Flores. No sanitary drainage system is installed in the area, the common practice is to use domestic septic tanks for black water disposal and discharge greywater to the stormwater drainage system. Greywater first runs on the surface of the street through the gutter, then enters the conventional stormwater drainage that discharges directly to the river without any treatment. Figure 5 shows an image of dry-weather runoff running through Siglo XXI. Solid waste collection service is also operated by the municipality of Flores and covers 100% of Siglo XXI, however, solid waste also enters the drainage system and ends up in the riverbank.

The municipality installed the existing stormwater drainage system more than two decades ago, when the area was urbanized. Unfortunately, the municipality does not keep any records or blueprints of the drainage system. Therefore, the information corresponding to the drainage system was gathered entirely during fieldwork. A dye-tracer analysis was carried out for the mapping of the drainage system. A red-coloured dye was discharged in the drainage inlets and through observation in the inspection wells, the pathway of the flow was determined. We repeated this procedure in all of the drainage system inlets to determine the layout of the existing infrastructure since no official information was available. The households’ greywater discharge pipes were georeferenced doing house-to-house observations.

#### Co-design factor 3: social acceptance (Rose 2020)

A brief questionnaire was prepared with the objective of gaining insights about how the residents of Siglo XXI perceive the idea of implementing Nature-based Solutions for greywater treatment in the neighbourhood. The questionnaire intended to collect qualitative information; the answers served to discriminate between favourable and non-favourable areas for the implementation of the experimental systems, the type of infrastructures preferred and provide hints on desirable design features.

Sixty residents were randomly interviewed, representing almost 20% of the properties in the neighbourhood. The survey was carried out during weekends, to facilitate finding residents at home with time to answer questions. Interviews were dynamic and conversational, showing pictures to facilitate comprehension of questions asked. Since residents might not be present at the time of the survey, or when present, might not be willing to participate, a systematic random sampling approach was implemented, selecting properties randomly while maximizing the cover area. Five questions were asked, taking approximately 15 minutes to complete each interview.

## Results

### Co-design process

#### Co-design factor 1: urban landscape configuration

**Fig. 6:**
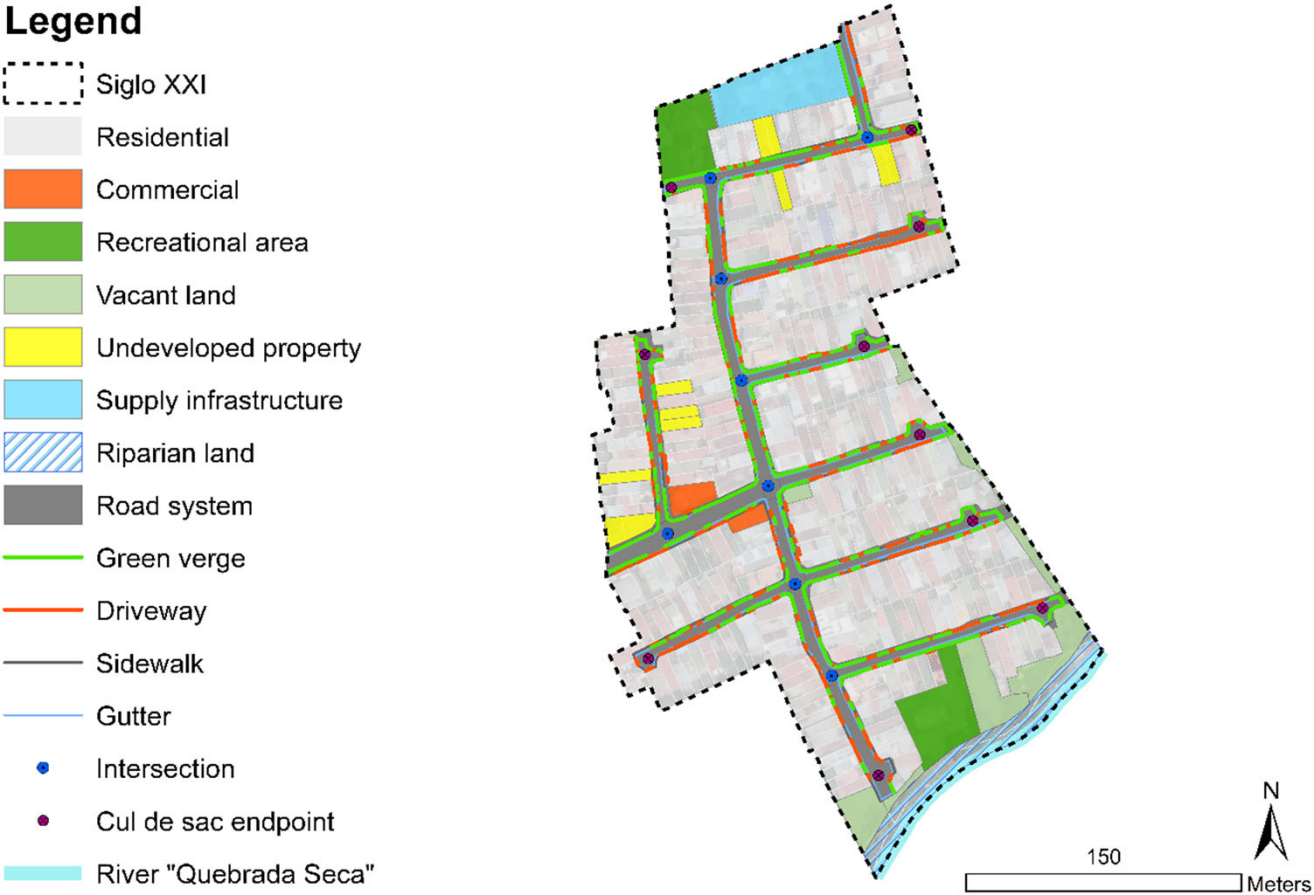
Classification of properties in the neighbourhood Siglo XXI and road components. Green verges along streets, supply infrastructure, recreational areas, and vacant lands shown in the map; are managed by the municipality. Source: (Fluhrer et al. 2021) based on cadastral information provided by the municipality and field observations and measurements

Potential implementation sites to retrofit Nature-based Solutions for greywater runoff treatment are existing green areas, which consist of various types of unsealed surfaces. The identification of green areas, road components, and the classification of properties is shown in Fig. 6. This identification was based on cadastral information provided by the municipality and field data collected through measurements and observations. The few existing undeveloped properties are privately owned and are planned to be developed into housing space.

Recreational areas such as playgrounds and sports facilities, vacant land, riparian areas, and other unsealed surfaces that compose the road system were considered as green areas, shown in Fig. 6. Along almost all the streets, there is an existing green network of green verges and roadside greenery, whose widths vary from 0.3 to 0.5 meters. There are two playgrounds designated as recreational areas at the north and south of the study area, and two vacant areas belonging to the municipality designated as public parks, near the riverbank. Along the riparian area of the river, a green corridor can be identified.

From this factor, it was determined that the implementation of any retrofit infrastructure is limited to public areas. Two main constraints were taken as inputs for the design: The current use and functionality of the public space must prevail. i.e. green verges along the streets are potential locations for treatment; however, these areas are interrupted by the passage of cars to the front yards of properties, which are used as a garage. The second constraint is that the design of public areas must obey national norms, dimensions and functions cannot be altered. Therefore, these aspects determined the placement of prototypes.

#### Co-design factor 2: dry-weather runoff drainage

**Fig. 7:**
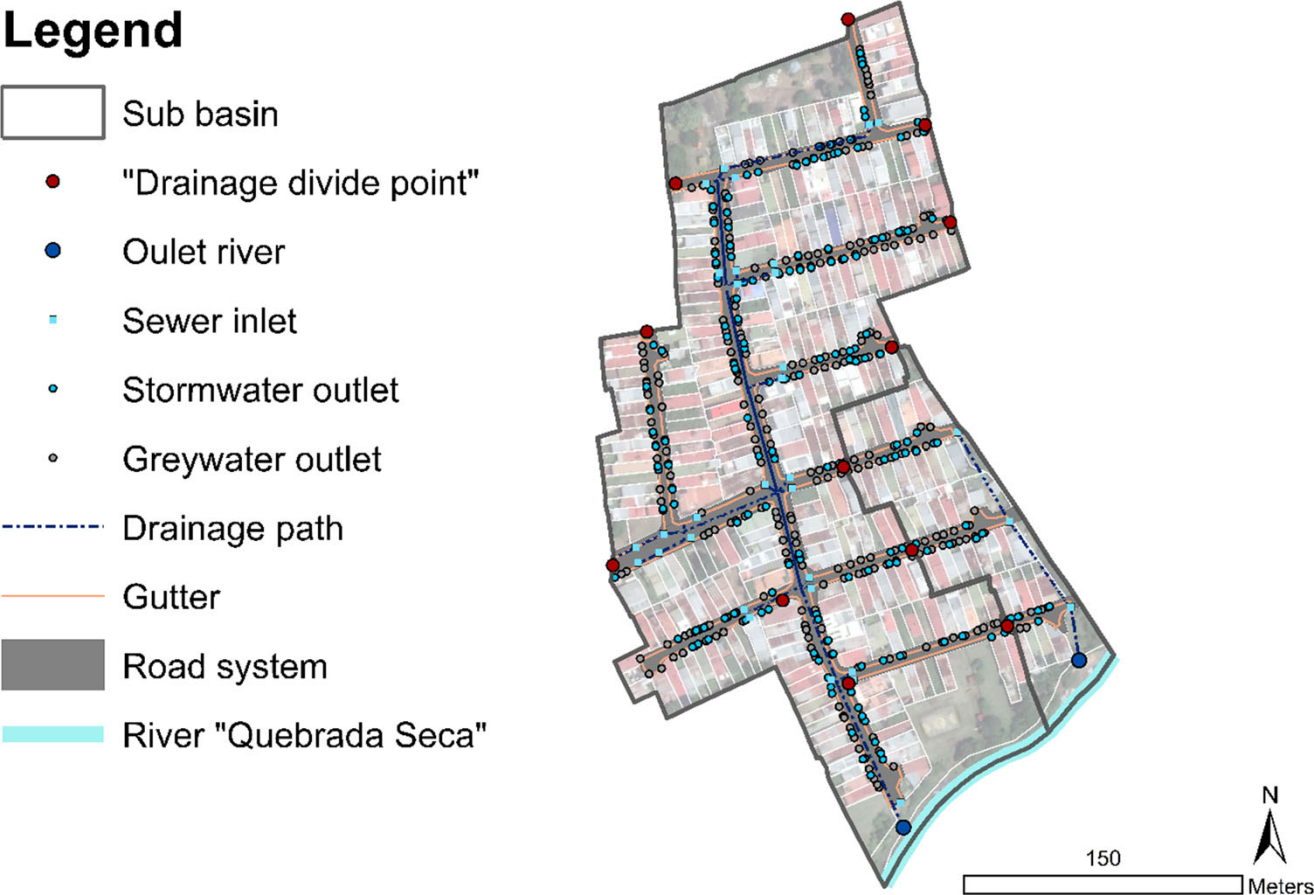
Stormwater drainage sub-basins in the neighbourhood Siglo XXI, drainage path, and discharge outlets to the river Quebrada Seca-Burio; Households’ greywater outlets to the gutter on streets are marked in grey dots. Source: Fluhrer et al. (2021)

The configuration of the existing drainage system, designed for stormwater conveyance, is depicted in Fig. 7. The image shows two stormwater drainage sub-basins in the neighbourhood and their outlet to the river Quebrada Seca-Burio. The location of every greywater outlet from households is referenced in the image (grey dots). The municipality registers an average water consumption of 20 m^3^ per household per month, from which, it is estimated that 80% is discharged as greywater (Instituto Costarricense de Acueductos y Alcantarillado 2017a, b), i.e. effluents from kitchens, showers, and sinks. There are 322 households in Siglo XXI, therefore a discharge of 172 m^3^ of greywater per day for the entire neighbourhood could be estimated. An ultrasonic sensor installed in the outlet pipe of the drainage system corresponding to the largest sub-basin registers maximum peak flow of 2 L s^−1^ of dry-weather runoff. From a characterization of greywater quantity and quality in the study area, it was determined a mean of 0.5 m^3^ per household; with an average BOD concentration of 205 mg L^−1^ (Rose 2020).

From the analysis of this factor the layout of the existing drainage infrastructure was mapped, its existence is considered an opportunity for design; the gutters and pipes could be used to collect and transport greywater to a decentralized treatment system. Therefore, this factor determined the final adapted-design of the prototypes.

#### Co-design factor 3: social acceptance

The results of the 5-question survey carried out in the neighbourhood is shown in Table 1. Questions 1 and 2 infer on the approval of potential changes of existing green areas and the perception about the impact of those changes in the current configuration of streets. Question 3 infers about resident’s consent regarding the implementation of greywater treatment system in front of their homes. Question 4 refers to the type of plants they would prefer in a nature-based treatment system, either ornamental (Heliconia) or non-ornamental; traditional constructed wetlands species that contribute to the removal efficiency of the system (i.e. Typha, Juncus, Phragmites); providing insights on design features of the treatment systems. The last question asks residents on their willingness to pay for the implementation of a system that treats their household greywater; it was also inferred in this question the willingness of residents to pay a monthly fee for maintenance.

**Table 1:** Questions asked to residents of Siglo XXI and the most frequent answers. Elaborated by the author, based on results from (Rose 2020)

Questions	Most frequent Answer	%
1. Would you like more green areas?	Yes	93
2. Would you accept a reduction in car designated areas in order to increase the green areas? (i.e. reducing the street area)	No	70
3. Would you accept that greywater infiltrates in the green area in front of your home? Do you like the idea that the greywater waters the plants?	Yes	77
4. Which plant do you like better? (Choose between two images: one showing traditional constructed wetlands species; the other shows one endemic ornamental species)	Heliconia (ornamental)	90
5. Would you be willing to contribute, hypothetically, a fixed amount of money each month for a one-year period? (i.e. to pay for the investments and maintenance on the treatment system)	*Average: 4.3 €/Month	

From this factor, it can be concluded that most neighbours are not willing to change the current design and use of the public space. Nevertheless, they would like to have more green areas around them. They also perceive greywater as a resource to improve greenery. Hence, it was considered as an opportunity for design that neighbours would approve to enhance green spaces by adding other functions such as the treatment of dry-weather runoff. This factor helped in determining the type of infrastructure with more acceptability, limiting the prototypes to only subsurface flow filtration systems.

### Knowledge integration for co-designed nature-based solution prototypes

The mechanism chosen to propose and experiment co-designed solutions is prototyping. Through the co-design process, constraints and opportunities that determined the type of infrastructure, final adapted-designs and location of placement for the prototypes were identified. A systematization of the analysis of the co-design factors and its outcomes in the exemplary study area is shown in Table 2.

**Table 2:** Systematization of the analysis of the co-design factors and its outcomes for the study area

Co-design factor	Analysed aspects	Determining aspect of design (implications)	Outcomes
Urban landscape configuration	• Analysis of public space • Existing green networks • Constraints due traffic • Current use and functionality of spaces	• Potential location for implementation NBS • Available areas • Type of NBS • Construction and safety regulations and standards	*Location of placement and sizing*: Implementation only in public spaces conserving actual areas i.e. green verges of streets and green area in riparian zone
Dry-weather runoff drainage	• Runoff pathway • Collection and transport of runoff • Identification of greywater discharges to gutters	• Optimal adaptation to the existing drainage configuration • Construction and safety regulations and standards	*Adapted design*: Adaptation of technical hydraulic features to profit from existing infrastructure i.e. inlet and outlets of prototypes
Social acceptance	• Perception of NBS in urban areas • Perception of greywater as a runoff • Willingness to change existing public space • Landscape design features • Type of vegetation preferred	• Type of NBS accepted • Location of NBS on private or public land • Type of vegetation • Landscape design	*Type of NBS*: Limited to subsurface filtration systems due to bad perception of surface flow (mosquito breeding & odours)

After carrying out the co-design process and analysing the factors in the study area, it was determined that different NBS could be implemented to treat greywater that flows through the gutters of the neighbourhood as dry-weather runoff. Finally, three prototypes were implemented distinguishing different scales for treatment, degree of centralization, and stakeholder involvement: household level, street level, and sub-basin level. For each of them, the analysis of the factors described above determined its design and functionality. The proposed prototypes were implemented with the cooperation and authorization of the Municipality and local dwellers. The construction was carried out by a private contractor and was funded completely by this research project. In the following sections, we describe the prototypes as an exemplification of the results derived from the application of the methodology in a real-life setting in a Central American context, with the aim of contributing to broader regional knowledge development and sharing.

#### Household level: horizontal subsurface flow constructed wetland installed in the sidewalk in front of the property

From the factors analysed during the co-design, we identified the potentiality to treat single households’ greywater before it is discharged to the gutter of streets. This treatment can be achieved by intercepting the main discharge pipe and use the area of the sidewalk in front of the property to install a treatment system. Therefore, this is proposed as a source control or household level of treatment. During the social analysis in the area, a particular family showed interest and willingness to accept this prototype to be installed in front of their property. The treatment system was designed based on guidelines for subsurface flow constructed wetlands, adapted to fit in the area available and to operate properly with the existing configuration of pipelines and urban layout.

**Fig. 8:**
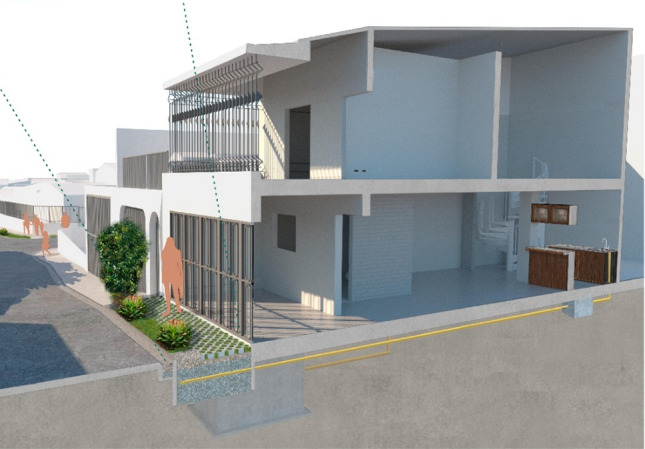
Design sketch of the subsurface flow constructed wetland installed in the sidewalk in front of the property. The wetland treats greywater discharge from the adjacent household. Source: sketched by Architect Laura Vargas under author’s guidance

The prototype consists of a small-scale subsurface horizontal flow constructed wetland installed in the area of sidewalk and green verge in front of the household property, the design sketch is shown is Fig. 8. The area of treatment is 5.2 m^2^, its dimensions are 1.5 m width, 3.5 m length with 0.6 m depth; corresponding to the entire available area in front of the property without affecting the street. The area was planted with ornamental species, i.e. *Heliconea* spp. This system is equipped with a pre-treatment grease trap installed in the pipe of the kitchen sink inside the property. The complete system is simple to operate and the owner of the property maintains it and ensures its functionality, by carrying out inspections and cleaning regularly.

#### Street level: bioretention area along sidewalk

The analysis of co-design factors in the study area showed the potentiality to modify green verges and sidewalks to use them as treatment areas for dry-weather runoff. The actual configuration of the drainage system conveys greywater to potential areas of treatment along the streets. The conceptual design of this solution is based on guidelines for bioretention areas or rain gardens, which are commonly used along streets or parking lots to detain stormwater by infiltration and at the same time, improve its quality. With the social analysis it was determined that the neighbours would accept the use of dry-weather runoff for irrigation of green areas to enhance greenery in public areas. Therefore, this prototype is proposed as a collective treatment for streets or blocks.

**Fig. 9:**
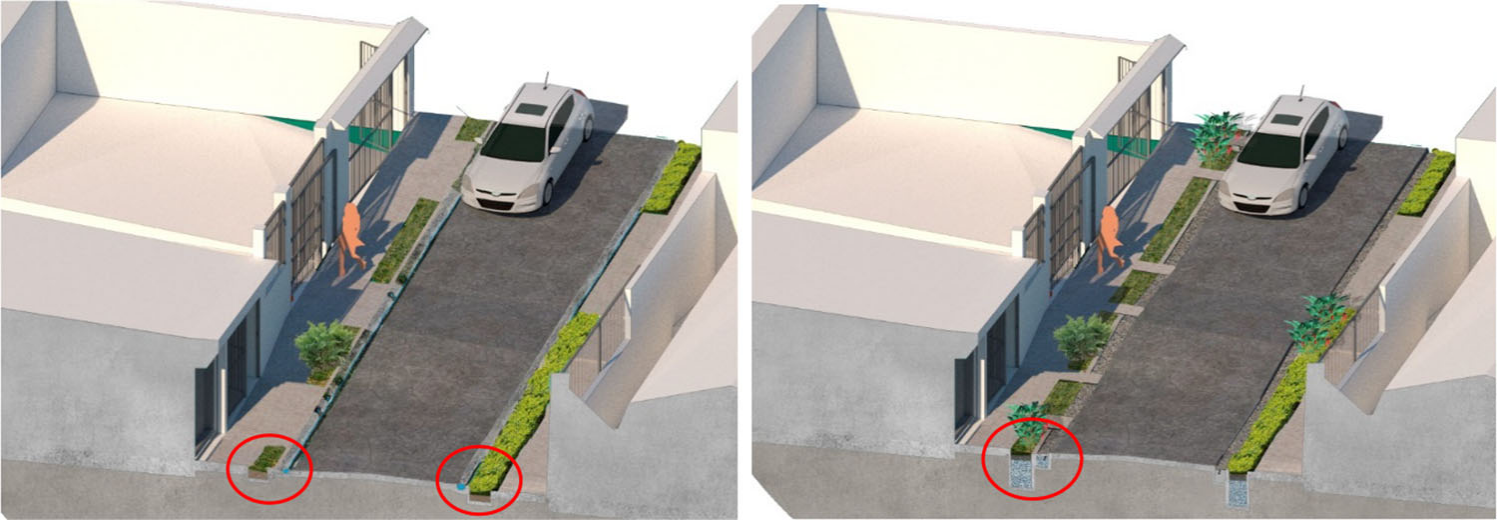
Sketches corresponding to prototype 2. The image on the left shows the current configuration of the sidewalk, curb and gutter. Dry-weather runoff is transported by the gutter to an inlet of the stormwater drainage system. The image on the right shows the modification of the green verge in the sidewalk into a bioretention area. After implementation, dry-weather runoff enters the bioretention area to be treated through filtration and then discharges to the existing drainage system. Source: sketched by Architect Laura Vargas under author’s guidance

The objective of this prototype is to treat greywater discharges from the households in one block of the neighbourhood. The infrastructure consists of a bioretention area with an underdrain pipe that discharges treated effluent to the stormwater drainage. The prototype was installed in the sidewalk and green verge of a residential street, the effective area of treatment is 20 m^2^, its dimensions are 2 m width, 10 m length with 0.6 m depth; corresponding to the entire available area of the sidewalk and green verge without affecting the street. The area was planted with ornamental species, i.e. *Heliconea* spp. A design sketch is shown in Fig. 9.

#### Sub-basin level: infiltration area

During the analysis of the existing drainage configuration and available green spaces in the neighbourhood, it was determined that the riparian area at the end of the pipe before the outfall of the drainage system to the river could be put to use for the treatment of dry-weather runoff. The challenge is to separate dry-weather runoff from stormwater runoff in order to implement an efficient greywater treatment system. Additionally, a key criteria had to be considered: the current use and function of the riparian area could not be changed. Therefore, only subsurface treatment systems could be considered, systems that allow that their surface is used for recreational greenery. The conceptual design of this prototype is based on an infiltration area with an underdrain distribution pipeline.

**Fig. 10:**
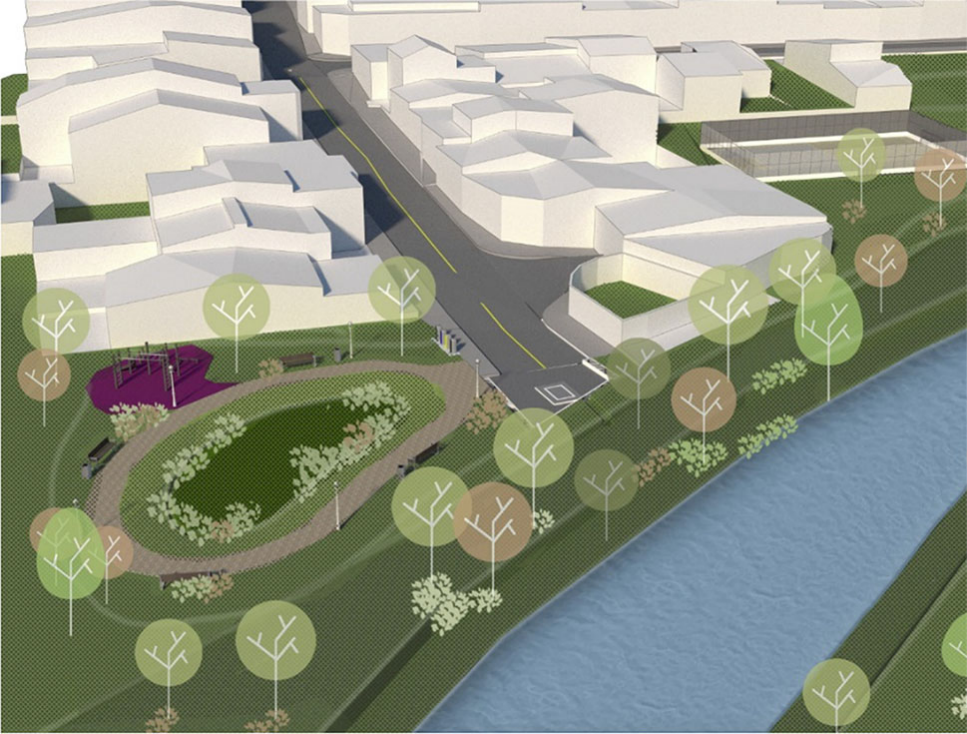
Design sketch of the infiltration area installed as an end-of-pipe treatment in a public green area adjacent to the river. The sketch depicts the area of infiltration to the left of the image and the pre-treatment sedimentation in the centre of the image. Source: sketched by Architect Laura Vargas under author’s guidance

The main function of this prototype is to treat the greywater discharge from the entire stormwater drainage sub-basin. The dry-weather runoff is collected at the outlet of the largest sub-basin of Siglo XXI, see Fig. 7. The infrastructure consists of an infiltration area, designed as a multifunctional element of a public park area owned by the Municipality. The area of treatment is 160 m^2^, with 1.5 m depth. The area was planted with two species of bamboo, i.e. *Gigantochloa atroviolacea, Bambusa oldhamii*. A pre-treatment is carried out in a two-chamber sedimentation tank; there the dry-weather runoff is collected and then distributed to the infiltration area. Design sketch of this prototype is shown in Fig. 10.

#### Comparison of prototypes and implications

The three implemented prototypes respond to different scale of treatment and opportunities to profit from current green spaces and drainage configuration. They also discriminate different stakeholders’ responsibilities; e.g. empowerment and ownership from the direct beneficiary was achieved in the case of the smallest scale of treatment. In the case of the larger scale, sub-basin level, the municipality takes responsibility. Table 3 presents a comparative summary of the experimental prototypes i.e. scale of treatment, investment costs and stakeholder involvement of the municipality and the neighbours.

**Table 3:** Summary of three prototypes that resulted from the co-design process implemented in the study area

Prototype	Scale of treatment (beneficiaries)	Investment Costs in USD	Operation & Maintenance requirements	Stakeholder involvement: Municipality	Stakeholder involvement: Neighbours
Horizontal subsurface flow constructed wetland	Household level (1 house)	2500	System works by gravity and natural processes. Cleaning the vegetated surface (carried out by the house owner)	Consult Municipality approved the modification of public space	Empower The owner of the property *Other neighbours were only informed
Bioretention area	Street level (15 houses)	11 000	System works by gravity and natural processes; cleaning and pruning the vegetated surface, removing solid waste from the system’s components	Partner Municipality is committed to maintain the prototype	Consult
Infiltration area	Sub-basin level (approx. 260 houses)	35 000			Involve Closest neighbours *Other neighbours were only informed

Stakeholder involvement was categorized using the Guidelines for co-designing and co-implementing Green Infrastructure in urban regeneration processes (Wilk et al. 2020)

Three prototypes were implemented in order to analyse them and draw conclusions on their effectiveness as well as their potentiality for replication or upscaling in the larger catchment area. During the next phases of this research project, the long-term performance, operation and maintenance costs and requirements, expected cost-benefit ratio, and provision of ecosystem services, will be analysed. The further understanding of these aspects will contribute to fill knowledge gaps needed to support the promotion of NBS in political and economic spheres; furthermore, the technical knowledge needed to improve understanding of how small-scale systems can address catchment-level objectives.

## Discussion

### Co-design process

This research was conducted under a transdisciplinary research project in the field of urban socio-ecology. We sought to ground our research to real-world problem solving by identifying local needs in the context of developing Latin America. Therefore, our methodology consisted on an adapted co-design process exemplified in a case study area in Costa Rica, that started by problem framing with local stakeholders. During the problem framing, we identified dry-weather runoff as a source of pollution to a river in a densely urbanized catchment, consequently, our problem solving focused on the treatment of this type of runoff before reaching the river. For that purpose, we aid the search of solutions by studying the application of sustainable stormwater treatment technologies in urban areas.

Ariza et al. (2019) proposed a methodology for the selection of stormwater management infrastructure in a case study area of Latin America with similar characteristics of the GAM in Costa Rica. They carried out a spatial analysis using satellite based and cadastral information, concluding that for the emplacement of SUDS, further site-specific analyses are necessary to reveal relevant social and technical aspects. They also found, that in residential zones, potential areas for SUDS are fractionated in small spaces, which corresponds to the scenario found in our study area; i.e. merely residential with 0.12 km^2^, where potential sites for implementation could be too small to be analysed exclusively with remote information. Given the goals of our research and the findings from Ariza et al. (2019), we consider that the methodology presented in this paper could be regarded as a follow-up to theirs; applicable when a microscale implementation is looked-for and further site specific analysis is needed.

Our methodology narrowed the analysis for the proposal of potential solutions to three co-design factors (1, 2, and 3 in Fig. 2). Two factors correspond to the biophysical characteristics and a third one corresponds to social aspects. The information regarding these factors was collected entirely in the field, the small scale and spatial fragmentation of the study area required the gathering of information strictly based on field observation, measurements and interviews. Therefore, this methodology required a significant amount of people involved in gathering the information in the field; which was possible due to the opportunity to have several students involved in the project. Nonetheless, if non-researchers would replicate this methodology, it would be needed to assign personnel working in the field collecting data, therefore this could be regarded as a limitation for its replication in other areas or contexts. On the other hand, we identified the potentiality for replication in community projects, in which there is active participation of beneficiaries who could easily collaborate in doing observation, measurements and interviews. Since there are no required qualifications needed for that purpose, the tasks could be assigned to interested neighbours or high school students. We consider this method could be of use for community project developers because it focuses on solving local specific problems with local solutions.

In the practical application of the co-design process, we highlight the social involvement as the main challenge. Main lessons learnt with regard to this topic in the real-life setting were: (a) there is difficulty in engaging people, raising and maintaining their interest in the process. (b) Interests vary drastically between the different stakeholders, even when people live in the same neighbourhood, the problems and priorities are not perceived homogenously. (c) We recognised that engaging the social community requires significant effort and resources, which can slow down the progress; coinciding with Polk (2015), who concludes that participatory and interactive research requires much more time than traditional approaches. On the other hand, Wilk et al. (2020) advise that engaging stakeholders early in the process helps to create a sense of ownership for the NBS and increase the chance of their maintenance and caretaking beyond termination of a pilot project. Therefore, for this research we alleged as imperative to involve the social community, as far as possible, given available time and resources. However, we recognize that in overall the community participation was much lower than desired, which could represent a weakness for the outcomes or the process itself.

A positive result of the attempt of engaging local stakeholders early in the process (i.e. workshops, meetings and divulgation sessions) is the contribution to improve the local understanding about urban ecology and the recognition of services provided by river ecosystems. It was also considered a positive outcome of the methodology, that the problem was framed by creating the setting for a co-diagnosis of the space of intervention as a self-reflection on how common day-to-day activities affect negatively the river ecosystem and from that perspective find ways to solve it, building consciousness about the impact of solutions. With this process, we achieved to raise empowerment of infrastructural solutions, although not entirely. We perceived that responsibilities or commitments for the long-term operation of NBS were preferably avoided by stakeholders; Sharma et al. (2016) also found reluctance to assume responsibility for the operation and maintenance of WSUD in Australia. This could be a reflection of knowledge gaps in the practice of NBS, there is still a prevalence of uncertainties around the actual implications related to operation and maintenance, overall costs and benefits, effective lifespan, responsibilities assignment, and perception of residents surrounded by these infrastructure (Sharma et al. 2012; Echavarria et al. 2015; Sharma et al. 2016; O’Donnell et al. 2017; Williams et al. 2019). In that sense, prototyping and post-implementation monitoring could contribute to the knowledge generation to overcome these uncertainties. Moreover, co-design processes with stakeholders engagement could identify fears related to these uncertainties and could tackle them early in the design stage.

Overall, our findings seem consistent with conclusions and recommendations of Polk (2015); Webb et al. (2018); Wilk et al. (2020), who analysed processes for transdisciplinary co-production of knowledge targeting real-life problem solving, highlighting the relevance of application of holistic approaches to have a better understanding of complex dynamic urban systems from a whole-system-view. When regarding NBS, Wilk et al. (2020) suggest that involving local experiential knowledge through the co-design process improves the design and implementation of NBS, creating solutions that respond to and are tailored to the local context, its challenges and the local communities’ needs. Williams et al. (2019), highlights the need to study social perception of SUDS and how designs could address residents’ concerns. With this methodology, the integration of different type and sources of knowledge contributed to propose tailored and adapted solutions that fit better in the local context, supporting the statement from Wilk et al. (2020) and Williams et al. (2019). Yet, more evidence need to be gathered during a post-implementation monitoring.

### Barriers and constraints for implementation of NBS in Latin America

Little information is available on experiences of design and implementation of NBS in urban areas of Latin America (Echavarria et al. 2015; Dobbs et al. 2019), though it may be recently emerging in a few countries (Romero-Duque et al. 2020). We described the case study in a real-life setting in the Central American context, with the aim of contributing to broader regional knowledge development and sharing. The co-design process and implementation of NBS for urban runoff management brought to light limitations, barriers and constraints for the realistic and successful implementation of NBS in the study area, which could resemble to other cities in developing countries of Latin America, hindering the adoption of this concept in the region.

An identified barrier relates to economic aspects i.e. low public investment; investments allocated to promote conventional technology to follow standards or trends from developed countries; stringent opportunities for innovation by rigid conventional methods of funding organizations. Additionally, lack of knowledge and experience of implementation of NBS in the region seems to endorse conventional technology in the socio-political sphere. A 2019 report from the Inter-American Developing Bank (Watkins et al. 2019) also acknowledged these limitations in the Latin American context.

Climatic conditions of tropical areas promote faster stagnant water decomposition and algae grow in blue/green infrastructure in urban areas. Additionally, these infrastructures are associated with mosquito breeding, increasing the occurrence of waterborne diseases, thus causing negative social perception (Williams et al. 2019) and even the rejection of this type of infrastructures, favouring the application of conventional grey infrastructure that quickly conveys water far away (Echavarria et al. 2015). Therefore, this aspect was considered a constraint to promote the adoption of some types of NBS in urban areas, although it is not proven that mosquito breeding would be promoted through them.

Technical constraints were also identified during this research. The lack of information or records regarding the existing public infrastructure made it challenging to retrofit new features in fully developed areas. With the co-design process proposed in this research, we aimed to narrow down to essential minimum requirements, easing the process of collecting information needed and speeding the progress into the implementation phase. From the experience, we identified that there could always remain gaps of information that could affect the progress of a retrofitting project, e.g. unexpected discoveries in the existing drainage layout during the construction phase in this project forced delays and last-minute changes in the configuration of prototypes.

Mismanagement of solid waste represents an additional burden to the existing drainage infrastructure; NBS cannot easily address this problem (de Bruijn 2015). Therefore, it results in an increase of maintenance requirements or negative effects in the operation and long-term integrity of NBS infrastructures; e.g. excessive solid waste in the inlet of prototypes built for this project causes deficiency in their operation. During the construction phase, it was identified that local professionals are unfamiliar with NBS construction; therefore, lacking the consciousness of expected goals and functionalities of the infrastructures to build. Roy-Poirier (2010) identified this limitation in the application of bioretention systems in Canada, Sharma et al. (2016) also reported on this constraint in Australia, whilst O’Donnell et al. (2017) identified the lack of knowledge from decision makers as a barrier in the UK.

Other aspect that could hinder NBS implementation in Latin America is the social-ecological particularity of cities in this region. Cities in Latin America are characterized by socioeconomic inequalities, reflected in segregated access to public services such as sanitation or green recreational areas (Dobbs et al. 2019). Correspondingly, intrinsic cultural perceptions and prejudices were identified during the analysis in this research, we acknowledged social inequalities even within the small neighbourhood that determine behavioural patterns e.g. trees/bushes in urban residential areas are perceived as dark and potentially dangerous zones, and therefore neighbours refuse them. Thus, supporting the need to develop co-design processes that promote different stakeholders inclusion, altogether problem framing and prioritization of needs.

Additionally, unwillingness to change or innovate the existing configuration of the urban matrix and its functionality was also identified during this research; similarly O’Donnell et al. (2017) found that reluctance to support novel approaches and change of practices counts as the most perceived barrier in the UK.

Most of the constraining factors identified during this research have been mentioned in literature from different parts of the world (Sharma et al. 2012; Sharma et al. 2016; O’Donnell et al. 2017; Dobbs et al. 2019). Suggesting that these factors could be limiting the extensive adoption of NBS, therefore, efforts need to be done in understanding context-specific aspects in the design and long-term operation of NBS. Co-design processes are useful to identify these context-specific aspects relevant for planning and designing. Though stakeholders’ involvement may be resource consuming, the integration of different sources of knowledge contribute to design tailored solutions that tackle prioritized needs. Prototyping is a useful method to contribute to knowledge generation regarding construction, operation, maintenance, costs, benefits and services, and long-term effectivity of NBS; that could help to overcome existing uncertainties around these aspects. We recommend the promotion of NBS prototypes that imperatively include post-implementation monitoring and assessments. Furthermore, the creation of spaces for knowledge sharing in the region, where the implementation experiences could be disseminated to other practitioners and researchers.

## Conclusions

This paper presents the practical application of a NBS co-design process in Central America in a structured and systematic manner, identifying from the beginning, particular factors that affect the NBS design and implementation process. This research contributes to fill knowledge gaps regarding the implementation process of NBS in developing countries, specifically in Latin America. The methodology presented in this paper intend to propose an adaptive method that explore ways for the implementation of NBS based on context-specific information and different knowledge sources. The consideration of only three main factors of the area of intervention sought that the process would be efficient, focusing resources on analysing essential data for the implementation. The application of a transdisciplinary co-design process pursued that solutions were not imposed by externals; final users and beneficiaries were included in the process, resulting in solutions that are presumably more adapted and fitted for higher probabilities of success. Engaging local dwellers was stated as imperative to carry out this research, though it resulted being the main challenge. In this experience, we seek to include the different stakeholders very early in the problem definition and solution design, as sources of knowledge and information that defined critical aspects of the designs.

This paper brings to light valuable insights that may be hindering the application of NBS in the region. Practical limitations and constraints were identified during the put-in-practice of the adaptive methodology. The co-design process intended to identify potential NBS retrofits for decentralized runoff treatment in urban areas. Results show that the adoption of NBS in the region should not rely exclusively on technical guidelines available; a detailed analysis of context-specific conditions is crucially needed, since these conditions could limit the acceptance and sustainability of NBS infrastructures. We recommend the promotion of NBS prototypes that imperatively include post-implementation monitoring and assessments; we consider it a useful method for knowledge generation that contributes to overcome existing uncertainties around NBS. Co-design processes could be of use in identifying context-specific aspects relevant for prototype planning and designing. We highlight the need to create opportunities for regional experience exchange and communication of progress in this field to improve knowledge needed to disseminate the implementation of NBS.
